# Does fasting during Ramadan increase the risk of the development of sialadenitis? 

**DOI:** 10.1186/s12903-020-01139-x

**Published:** 2020-05-29

**Authors:** Michael V. Joachim, Yasmine Ghantous, Suleiman Zaaroura, Kutaiba Alkeesh, Tameem Zoabi, Imad Abu el-Na’aj

**Affiliations:** 1 Department of Oral & Maxillofacial Surgery, Baruch Padeh Medical Center, Poriya, Israel and Azrieli Faculty of Medicine, Bar-Ilan University, Safed, Israel; 2 Department of Oral & Maxillofacial Surgery, Baruch Padeh Medical Center, MP Lower Galilee, Poriya, 1520800 Israel; 3 Department of Otolaryngology, St. Vincent de Paul (French) Hospital, POB 50294, Nazareth, 1616102 Israel

**Keywords:** Adenitis, Salivary gland, Islam

## Abstract

**Background:**

Ramadan is a month within the Islamic lunar calendar when Muslims are required to fast (abstain from food and drink) during the daytime (from sunrise to sunset) for the entire month. Due to the established connection between fasting and dehydration and acute sialadenitis, the aim of this study is to determine if there is a higher frequency of sialadenitis among the Muslim population during Ramadan than during other months of the year.

**Methods:**

We conducted a retrospective study using the medical records of 120 Muslim patients admitted to the emergency room (ER) and diagnosed with acute sialadenitis over a 5-year period at the Baruch Padeh Medical Center, Poriya, and St. Vincent de Paul (French) Hospital, Nazareth, both located in Israel. The study group were Muslim patients, with the aforementioned diagnosis, admitted during Ramadan, while the control group included patients diagnosed with sialadenitis during the rest of the year. We analyzed overall admission frequency as well as descriptive and diagnostic data, including age, sex, gland involved and several blood test results.

**Results:**

During the month of Ramadan, the admission of Muslims with a diagnosis of acute sialadenitis was more than double that during the other months of the year – a difference that was found to be statistically significant (*p* = 0.001). Additionally, we found that Ramadan sialadenitis patients had significantly higher leukocyte numbers at admission (*p* = 0.0085) and, importantly, a significantly higher level of dehydration (blood urea nitrogen (BUN)/creatinine ratio) than non-Ramadan sialadenitis patients (*p* = 0.0001).

**Conclusion:**

There is evidence that fasting in Ramadan may increase the risk for the development of acute sialadenitis. Our results suggest that this may be the result of dehydration.

## Background

Ramadan is the ninth month within the Islamic lunar calendar, and during Ramadan, Muslims are required to fast (abstain from food and drink) during the daytime (from sunrise to sunset) for the whole month [1]. Ramadan fasting represents a specific form of fasting, consisting of alternating fasting and feasting (re-feeding) periods [2]. Being based on the lunar calendar, the daily fasting duration varies depending on the period of the year and the latitude of the location [3].

There are several previous reports about metabolic changes related to fasting, such as weight loss and dehydration [4, 5]. Those changes may primarily affect individuals with diabetes [6], but there are also reports that Ramadan fasting may cause hypertension [7] and increase the incidence of ischaemic stroke [8]. However, for healthy individuals, the metabolic changes that occur are unlikely to have significant harmful consequences [9, 10]. Infirm individuals are waived from this religious duty; however, patients with various health issues might choose to share this event with peers and family members [11].

Acute sialadenitis is an inflammation of the salivary glands as a response to bacterial infection. The stasis of salivary flow and secretion as a consequence of dehydration or decreased oral water intake allows retrograde bacterial migration into the gland parenchyma [12]. In most cases, acute sialadenitis affects one major salivary gland, with the parotid being the most prevalent [13–15]. Previous reports found that the incidence of acute sialadenitis is 27.5 cases per million individuals [16].

The affected gland is swollen and painful, and the overlying skin may be warm and erythematous. An associated low-grade fever and trismus may be present. A purulent discharge is often observed from the duct orifice when the gland is massaged [12–14].

Dehydration can be assessed through blood hydration status markers on blood tests, especially the blood urea nitrogen (BUN)/creatinine ratio [17, 18].

Given the connection between dehydration and acute sialadenitis, we hypothesized that there is a higher frequency of sialadenitis among the Muslim population during Ramadan than during other months of the year. This study, to the best of our knowledge, is the first study investigating the connection between Ramadan and the incidence of acute sialadenitis, and its objective is to bring this phenomenon to public awareness as a by-product of the religious obligation.

## Methods

This study was approved by Poriya Medical Center Institutional Review Board (approval # POR-18-0061) and was performed in accordance with the Declaration of Helsinki, seventh revision (2013). This is a retrospective study prepared according to STROBE guidelines. The hospitals involved in this study are uniquely positioned to address the hypothesis that Ramadan fasting corresponds to an increased frequency of acute sialadenitis since a large portion of their patient base are Muslim. Baruch Padeh Medical Center is a public hospital, run by the Israeli Ministry of Health, serving the area of Eastern Galilee and Golan Heights and is a multi-regional centre for oral and maxillofacial surgery. It is estimated that 40–60% of its patients come from the Muslim community. St. Vincent de Paul (French) Hospital is a public hospital, run by the Catholic Church Trust, located in the heart of Nazareth, the largest Arab city in Israel, and provides exclusive otolaryngology service in that area. More than 80% of its patients come from the Muslim community.

The research sample included all adolescent and adult (> 14 yrs) cases of acute sialadenitis (identified by the International Classification of Diseases, Ninth Revision (ICD-9) code 527.2 – Sialadenitis) diagnosed in Muslims (data on patient religion was received from the Israeli Ministry of Interior database) in the emergency rooms of Baruch Padeh Medical Center, Poriya and St. Vincent de Paul (French) Hospital, Nazareth (both affiliated with the Azrieli Faculty of Medicine, Bar-Ilan University, Safed, Israel and located in the northern part of Israel) during the Hijri years 1434–1438 (15/11/2012–20/09/2017). The age filter was chosen to increase the probability of fasting and to rule out juvenile conditions. Patients with other conditions that might lead to acute dehydration (diarrhea, gastroenteritis, hyperemesis) were excluded from the analysis. Pregnant women are exempt from fasting and thus were not included in this study.

For this sample (sialadenitis diagnosis, Muslim, > 14 years age) we calculated the frequency of ER admissions due to acute sialadenitis for each Hijri month in the timeframe and compared Ramadan months to other months of the year. For the sake of accuracy, analysis was carried out based on Hijri calendar months since the month of Ramadan is part of this lunar calendar which is 11 days shorter than the standard Gregorian solar calendar. It is important to note that we cannot be sure of the fasting status of each patient, but given that Ramadan fasting is one of the main pillars of Islam and is practised by the vast majority of Israeli Muslims we assumed that the majority of the patients included in this study were indeed fasting during the month of Ramadan.

Additionally, in order to exclude the possibility of comorbidities whose prevalence may rise in an older population, we assessed for significant differences in the mean age of patients admitted during Ramadan and during other months of the year.

Statistics were calculated using Microsoft Excel™ (2010 version, Redmond, WA, USA) and IBM SPSS™ (25.0 version, Armonk, NY, USA) software. The comparison between subgroups was made using the following non-parametrical tests: ANOVA, t test, Kruskal-Wallis test, Wilcoxon signed-rank test, Fisher’s exact test and Mann-Whitney U test. Adjustment factors were calculated by Pearson’s method. A *p* value < 0.05 was considered significant.

## Results

**Fig. 1:**
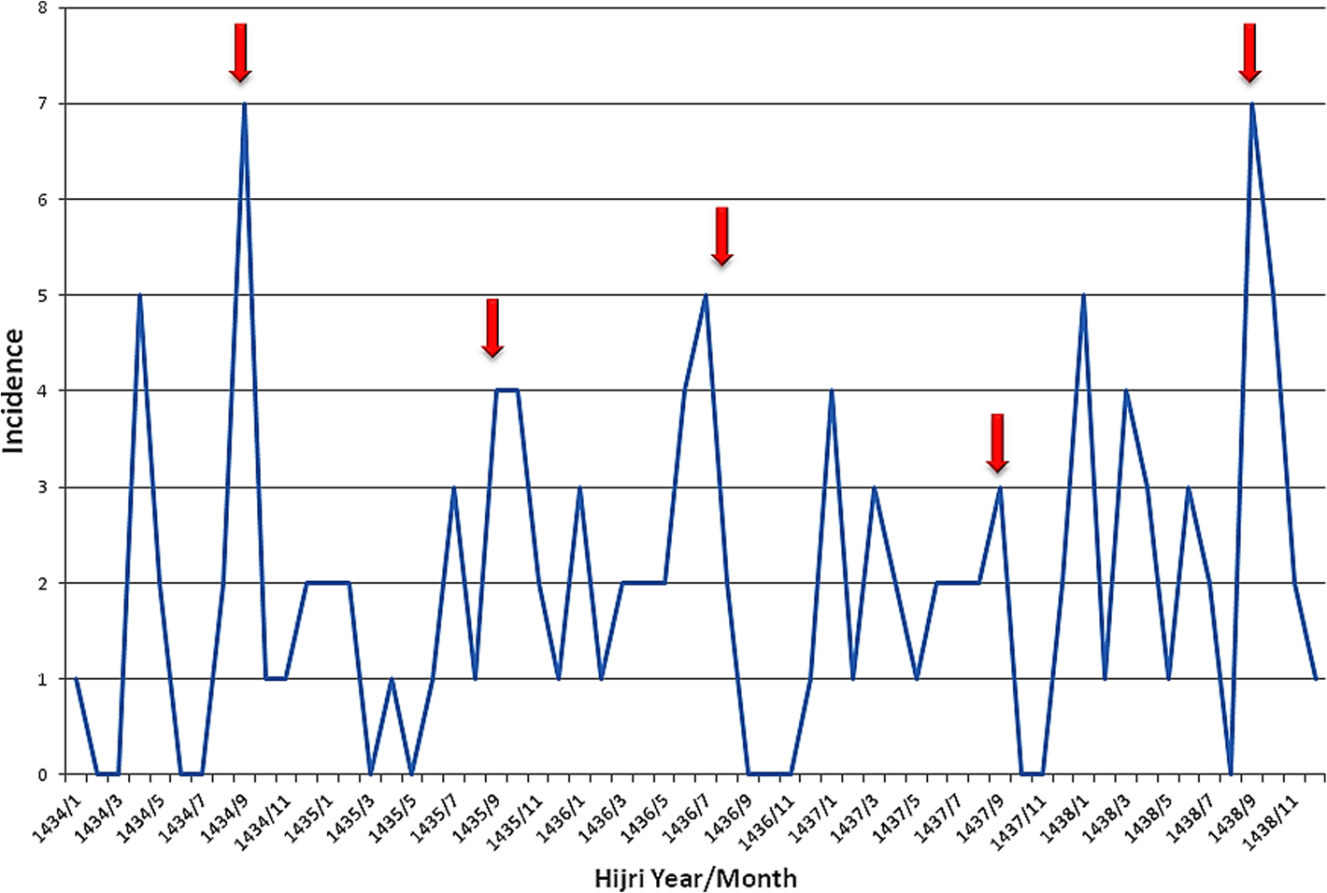
Prevalence of sialadenitis during the study

Our data showed that 21 Muslims were admitted to the ER due to acute sialadenitis in the aforementioned hospitals during the 5 Ramadan months in the timeframe of the study, revealing an incidence of 4.2 cases/month (95% CI 2.641–6.176). This incidence was high compared to that during non-Ramadan months: 99 admissions due to acute sialadenitis in 55 non-Ramadan months, resulting in an incidence of 1.8 cases/month (95% CI 1.471–2.166). The risk ratio (RR) of ER admission due to acute sialadenitis during Ramadan was 2.33 (95% CI 1.46–3.72) and was statistically significant (*p* = 0.001). The curve for the prevalence of acute sialadenitis across months is presented in Fig. 1.

The variables and statistical analyses are detailed in Table 1. A comparison of blood test results from Ramadan vs. non-Ramadan sialadenitis patients revealed that Ramadan sialadenitis patients had significantly higher mean leucocyte numbers [*10^9^/L] (8.98 ± 2.75 vs. 6.08 ± 4.78, *p* = 0.0085) and significantly higher mean creatinine [mg/dL] (0.81 ± 0.33 vs. 0.62 ± 0.13, *p* = 0.0001) and mean BUN [mg/dL] (14.26 ± 3.85 vs. 5 ± 2.08, p = 0.0001) levels. The combined mean BUN/creatinine ratio was significantly higher in Ramadan sialadenitis patients (17.7 vs. 8.06, p = 0.0001). There was no difference in amylase rates between Ramadan and non-Ramadan sialadenitis patients [U/L] (360 ± 405.25 vs. 370.25 ± 398.81, *p* = 0.915).

**Table 1:** Research data and statistical analysis (*N* = 120)

		Ramadan Sialadenitis Patients	Non-Ramadan Sialadenitis Patients	*p*-value	95% CI
Total Patients		21	99	N/A	N/A
Ratio/month		4.2	1.8	0.001^a^	1.46–3.72
Age	Mean	42.88	42.88	1^b^	(−10.09)-10.09
	SD	16.71	22.02		
Sex	Male	13	57		
	Female	8	42		
	M:F ratio	1.62	1.36	0.71^a^	0.52–1.94
Gland	Parotid	12	58		
	SM	9	41		
	P:SM ratio	1.28	1.4	0.86^b^	0.23–1.95
Leukocytes (*10^9/L)	Mean	8.98	6.08	0.0085^b^	0.76–5.04
	SD	2.75	4.78		
Amylase (U/L)	Mean	360.00	370.25	0.915^b^	(−200.49)-179.99
	SD	405.12	398.81		
BUN (mg/dL)	Mean	14.26	5.00	0.0001^b^	8.08–10.43
	SD	3.85	2.08		
Creatinine (mg/dL)	Mean	0.81	0.62	0.0001^b^	0.104–0.28
	SD	0.33	0.13		
BUN/Cr		17.70	8.06	0.0001^a^	2.46–3.28

*Abbreviations: SD* Standard deviation, *SM* Submandibular, *P* Parotid, *BUN* Blood urea nitrogen, *Cr* Creatinine, (*d*) *L* (deci)Liter, (*m*)*g* (milli)gram, *U* Units

^a^ Chi-squared test, ^b^ t test

The mean age of Ramadan sialadenitis patients was 42.88 ± 16.7 years (SD, range 17–71), comparable to non-Ramadan sialadenitis patients whose mean age was 42.89 ± 22.02 years (SD, range 14–88). Additionally, there was no significant difference in M:F ratio or gland type affected between the two groups (Table 1).

## Discussion

The results of this study show that the frequency of ER admissions due to acute sialadenitis significantly increased during Ramadan compared to non-Ramadan months over a multi-year analysis. Although, the authors were not able to find any previous report associating Ramadan and salivary gland disorders, these results correspond with the known connection between acute sialadenitis and dehydration.

In most cases, acute sialadenitis affects one major salivary gland, with the parotid being the most prevalent [13–15], and the prevalence of acute sialadenitis increases in medically compromised, hospitalized, or postoperative patients [19]. However, in this study the prevalence of parotitis was lower than that of submandibular sialadenitis during Ramadan. This trend in prevalence was present in a few previous reports [20]; however, it is difficult to draw conclusions from this observed trend since the study group is small and the differences here were not statistically significant.

Another inciting etiology of acute sialadenitis is retrograde bacterial contamination from the oral cavity [15, 21]. Predisposing factors for the ductally ascending infection are dehydration, xerogenic drugs and salivary gland diseases associated with ductal obstructions or reduced saliva secretion [12, 22]. Other factors include hypothyroidism, renal failure, HIV, diabetes mellitus and Sjögren syndrome [15].

As previously mentioned, patients suffering from acute sialadenitis present pain and swelling of the affected gland, which may be accompanied by the expression of pus from the respective intraoral orifice, requiring antibiotic therapy directed by culture of the pus.

The management of the condition involves treating the infection and reversing the underlying medical condition and predisposing factors [12, 19]. Salivary flow stimulation by hydration is highly important, as well as the application of warm compresses, salivary gland massage, the administration of sialagogues such as lemon drops or vitamin C lozenges and oral hygiene [23–25]. The recommended initial empiric antimicrobial therapy is directed at gram-positive (most commonly *Staphylococcus aureus*) [26–28] and anaerobic organisms with the use of augmented penicillin that contains beta-lactamase inhibitors (e.g., amoxicillin-clavulanate) to help in the treatment of penicillin-resistant bacteria [19, 26–28]. Other options include clindamycin, cefoxitin, imipenem, and the combination of metronidazole and a macrolide [27]. Culture-directed therapy is also possibly administered. Rarely, acute suppurative sialadenitis can lead to abscess formation; in those cases, surgical drainage is indicated. Rarely, acute suppurative sialadenitis can lead to abscess formation; in those cases, surgical drainage is indicated [19].

The significant difference in the BUN/creatinine ratio between Ramadan and non-Ramadan sialadenitis patients and the fact that Ramadan sialadenitis patients presented dehydration hint that there is an association between fasting and an increased risk for acute sialadenitis. It is important to mention that the BUN/creatinine ratio is considered abnormal at a value > 20:1; however, the difference between the groups is important and significant and shows that the study group was less hydrated than the control group.

The analysis of leukocyte count uncovered an interesting phenomenon; both groups presented normal leukocyte counts (although that of the Ramadan sialadenitis group was slightly higher) that were “left shifted”, suggesting a bacterial infection. The Ramadan sialadenitis group seemed to have the more serious condition, created by dehydration. This finding is also supported by a systematic review showing that patients suffering from any condition that heightens the risk of the development of infectious complications should not fast [29].

These results of this study support our hypothesis and, assuming all other predisposing factors stay the same year-round, we conclude that there may be causality between Ramadan fasting (and subsequent dehydration) and an increase in the incidence of acute sialadenitis.

We suggest that physicians should also consider the patient’s eagerness, since religion fosters positive psychosocial outcomes and reinforces treatment adherence and compliance in Muslim patients [1, 29].

Thus, in the case of no medical restriction, fasting should not be discouraged in Muslim patients who are enthusiastic about Ramadan fasting. Physicians should be aware of this risk, and patients should be instructed to recognize some warning symptoms.

This study has a few limitations. First, it is a retrospective study; thus, it was assumed that all of the patients were fasting during the month of Ramadan, but this was not confirmed. It will be useful to conduct a prospective study with documented fasting status (if ethically possible). Second, with this study design, it is difficult to establish causality; rather, only any association between Ramadan and sialadenitis incidence can be determined. Finally, it will be useful to investigate a larger sample over a longer period in order to strengthen the findings in this study.

## Conclusion

Our study indicates that fasting (and subsequent dehydration) in Ramadan may increase the risk for the development of acute sialadenitis. The Muslim population and their physicians should be made aware of this risk in order to provide fast and effective treatment.

## Data Availability

The datasets used and/or analyzed during the current study are available from the corresponding author on reasonable request. The data is was collected and stored in Hebrew and English and is courtesy of two different Medical Centers – it’s sharing with public repositories was not approved by the IRB neither MC managements.

## Abbreviations

ANOVA: Analysis of Variance
BUN: Blood Urea Nitrogen
CI: Confidence Interval
ER: Emergency Room
ICD-9: International Classification of Diseases, Ninth Revision
MC: Medical Center
MS: MicroSoft
SD: Standard Deviation
SPSS: Statistical Package for the Social Sciences
